# An Efficacy and Feasibility Analysis of Chinese Patent Medicine Combined With Letrozole in the Treatment of Women With Ovulation Disorders: A Network Meta-Analysis

**DOI:** 10.3389/fphar.2021.722122

**Published:** 2021-10-05

**Authors:** Jing-Yan Zhu, Jian Liu, Xiao-Jing Cao, Xiao-Yun Wang

**Affiliations:** ^1^ Department of Gynecology, The Second Clinical Medical College of Guangzhou University of Chinese Medicine, Guangzhou, China; ^2^ Department of Gynecology, Guangdong Hospital of Chinese Medicine, Guangzhou, China

**Keywords:** Chinese patent medicine, letrozole, ovulation disorder, network meta-analysis, guiding clinical practice

## Abstract

**Objective:** To compare the efficacy of various listed Chinese patent medicines combined with letrozole in the treatment of ovulation disorders using network meta-analysis (NMA). **Methods:** We conducted a systematic literature search in PubMed, Cochrane Central Register of Controlled Trials, Embase, Chinese Biomedical Literature, China National Knowledge Infrastructure, Wanfang, and VIP Information databases up to June 2020. Randomized controlled trials reporting Chinese patent medicine combined with letrozole for ovulation disorders were included. The Stata 13 and WinBUGS1.43 software were used for data analysis. **Results:** A total of 24 randomized controlled trials were included, involving 2,318 patients. The results showed that when compared with patients using only letrozole, the ovulation rate was higher in patients using letrozole combined with Kuntai capsules, Fuke Zaizao capsules, Fufang Xuanju capsules, or Dingkun Dan, and Fufan Xuanju capsules showed the greatest improvement; the pregnancy rate was higher in patients using letrozole combined with Kuntai capsules, Fuke Zaizao capsules, or Dingkun Dan; and the endometrial thickness on the day of follicular maturity was greater in patients using letrozole combined with Kuntai capsules, Fuke Zaizao capsules, Fufang Xuanju capsules, Bailing capsules, or Dingkun Dan. In terms of the sequencing of NMA results, Fufang Xuanju capsules combined with letrozole gave the best results in improving the ovulation rate and increasing the endometrial thickness, while Dingkun Dan combined with letrozole achieved the best results for improving the pregnancy rate. **Conclusion:** Letrozole combined with Chinese patent medicine is more effective than letrozole alone in the treatment of ovulation disorders. Fufang Xuanju capsules is good at improving the ovulation rate and increasing the endometrial thickness. Dingkun Dan is good at improving the pregnancy rate. The appropriate choice of treatment should be made according to the actual clinical situation. This study is registered with the International Prospective Register of Systematic Reviews (CRD42020200603).

## Introduction

Ovulation disorders are a common cause of infertility or difficulty in achieving pregnancy in women of childbearing age. In infertile women, approximately 25–30% of patients have ovulatory dysfunction. (Xie et al., ) The induction of ovulation is the main treatment protocol for women with ovulation disorders who require fertility treatment. Letrozole is an aromatase inhibitor that can inhibit the transformation of androgen to estrogen and reduce the estrogen level. The negative feedback acts on the pituitary gland to encourage the secretion of gonadotropin and stimulate the development of the follicles. Letrozole is currently a commonly used clinical drug and has started to replace clomiphene as a first-line medicine. (Vitek and Hoeger, 2019) In gynecology or reproductive specialty departments of many Chinese and Western medicine hospitals in China, doctors will combine letrozole with gynecological Chinese patent medicine to promote the ovulation effect.

Many clinical studies have proven that a combination of Chinese patent medicine and letrozole in the treatment of ovulation disorders is more effective than using letrozole alone, and the combination of these drugs can increase the ovulation and pregnancy rates of patients and improve endometrial receptivity. Some of these studies have included meta-analyses of the combined treatment of some Chinese patent medicines and letrozole. However, there are currently few studies that have compared the efficacy of different Chinese patent medicines, making it difficult to evaluate the efficacy of various Chinese patent medicines when combined with letrozole in the treatment of ovulation disorders. Network meta-analysis (NMA) can summarize the existing evidence through direct comparisons, provide useful information through indirect comparisons, and rank the effects of various interventions to provide insights into the advantages and disadvantages of these interventions.

Therefore, this study uses the NMA method to systematically evaluate the efficacy of various Chinese patent medicines when combined with letrozole in the treatment of ovulatory disorders to provide evidence for clinical treatment.

## Methods

### Protocol and Registration

This research followed the Preferred Reporting Items for Systematic Reviews and Meta-Analyses for the Network Meta-Analysis checklist. (Hutton et al., 2015) The protocol was registered on the International Prospective Register of Systematic Reviews (registration number CRD42020200603).

### Inclusion and Exclusion Criteria

We used the following inclusion criteria:

1) Participants: patients with ovulatory disorders (e.g., ovulatory infertility and polycystic ovary syndrome) requiring fertility treatment
2) Intervention: any kind of Chinese patent medicine combined with letrozole
3) Comparison: letrozole alone
4) Outcomes: change in ovulation rate, pregnancy rate, and endometrial thickness after the treatment
5) Study design: randomized clinical trial (RCT)
6) Language: English or Chinese studies

We excluded studies based on the following criteria:

1) studies not including the required outcomes
2) repeated publications
3) self-control studies and non-RCTs
4) preclinical studies, systematic reviews, case reports, and meta-analyses
5) protocols, unpublished studies, or duplicate studies
6) studies based on a research design with obvious defects or suspected fraud

### Information Sources and Search Strategy

The Chinese Biomedical Literature, China National Knowledge Infrastructure, Wanfang, Weipu, PubMed, Embase, and Cochrane Central Register of Controlled Trials databases were searched to find RCTs on the use of Chinese patent medicine combined with letrozole in the treatment of ovulatory disorders.

Search words included Chinese medicine, patent medicine, herbal medicine, letrozole, Kuntai, Dingkun Dan, Fuke Zaizao, Bailing, Xiaoyao, Fuke Yangying, Fufang Xuanju, etc. The publication dates included were from the creation of each database to June 2020 (see Supplementary Table S1 for an example of the results retrieved from the PubMed database)

### Literature Screening and Data Extraction

Literature screening and data extraction were carried out independently by two researchers. First, the literature titles were imported into NoteExpress, and duplicates were identified. Second, according to the inclusion criteria, two researchers read the literature titles and abstracts and excluded those that did not obviously meet the inclusion criteria. Finally, the full text of the literature was obtained and read to further exclude those that did not meet the inclusion criteria or met any of the exclusion criteria.

In the literature that was finally included, the data were extracted from the studies using a premade data extraction table that included the study code, patient information, baseline and comparable data, intervention and control measures, treatment course, and outcome indicators.

### Quality Evaluation

Two researchers independently used the Cochrane risk of bias tool, as described in the Cochrane Handbook for Systematic Reviews of Interventions, to assess the quality of the RCT. Bias risks for each study were assessed based on six factors: random sequence generation, allocation concealment, blinding (performance and detection bias), incomplete outcome data (attrition bias), selective reporting (reporting bias), and other bias, and they were ranked as high, low, or unclear risk. If there were differences of opinion, they were solved through discussion or with the assistance of a third party.

### Statistical Analysis

#### Direct Pairwise Meta-Analysis

The ovulation and pregnancy rates are dichotomous variables, and the odds ratio (OR) was used to indicate the size of the effect. The endometrial thickness on the day of follicular maturity is a continuous variable, and the mean difference (MD) was used to indicate the quantity of the effect, and the 95% confidence interval (95% CI) of the OR and the MD were calculated. The RevMan 5.3 software was used for bias evaluation and direct pairwise meta-analysis. (Higgins et al., 2003)

Heterogeneity was quantitatively determined using I^2^, with I^2^ < 50% and *p* > 0.1 indicating no statistical heterogeneity. A fixed-effect model was used for meta-analysis. If statistical heterogeneity was found between the studies, subgroup and other methods were used to find the source of the heterogeneity, and if it could not be found, the random-effect model was used for meta-analysis. (Ades et al., 2006) A level of *α* = 0.05 was considered to be statistically significant.

### Network Meta-Analysis

The Stata 13.0 software was used to create the evidence diagram for the NMA.

We conducted an NMA to estimate the effect for each class and for each individual intervention using the Markov chain Monte Carlo methods in WinBUGS (version 1.43, MRC Biostatistics Unit, Cambridge, United Kingdom). (Lu and Ades, 2004) Two chains with different initial values were run simultaneously to assess convergence using Brooks–Gelman–Rubin diagnostic plots. We used Markov chains for 50,000 simultaneous iterations after the first 5,000 iterations were discarded because they may have had an influence on the arbitrary values. The iteration history diagram was drawn to evaluate the degree of convergence of the model. The deviance information criterion (DIC) between the fixed-effect model and the random-effect model was used to judge the degree of fit of the model. (Dias et al., 2013) A difference in the DIC of <5 indicated a consistent fit between the two models, and therefore, both models could be adopted. If the difference was >5, the model with the smaller DIC was adopted. When there was a closed loop, the consistency between the direct and indirect comparison was determined by the inconsistency factor (IF) value. When the starting point of the 95% CI of the IF value was zero, the direct and indirect evidence were considered to be consistent. Finally, evidence of the small-sample effect in the network was identified by drawing a comparison-correction funnel plot.

## Results

### Literature Retrieval Process and Results

**FIGURE 1:**
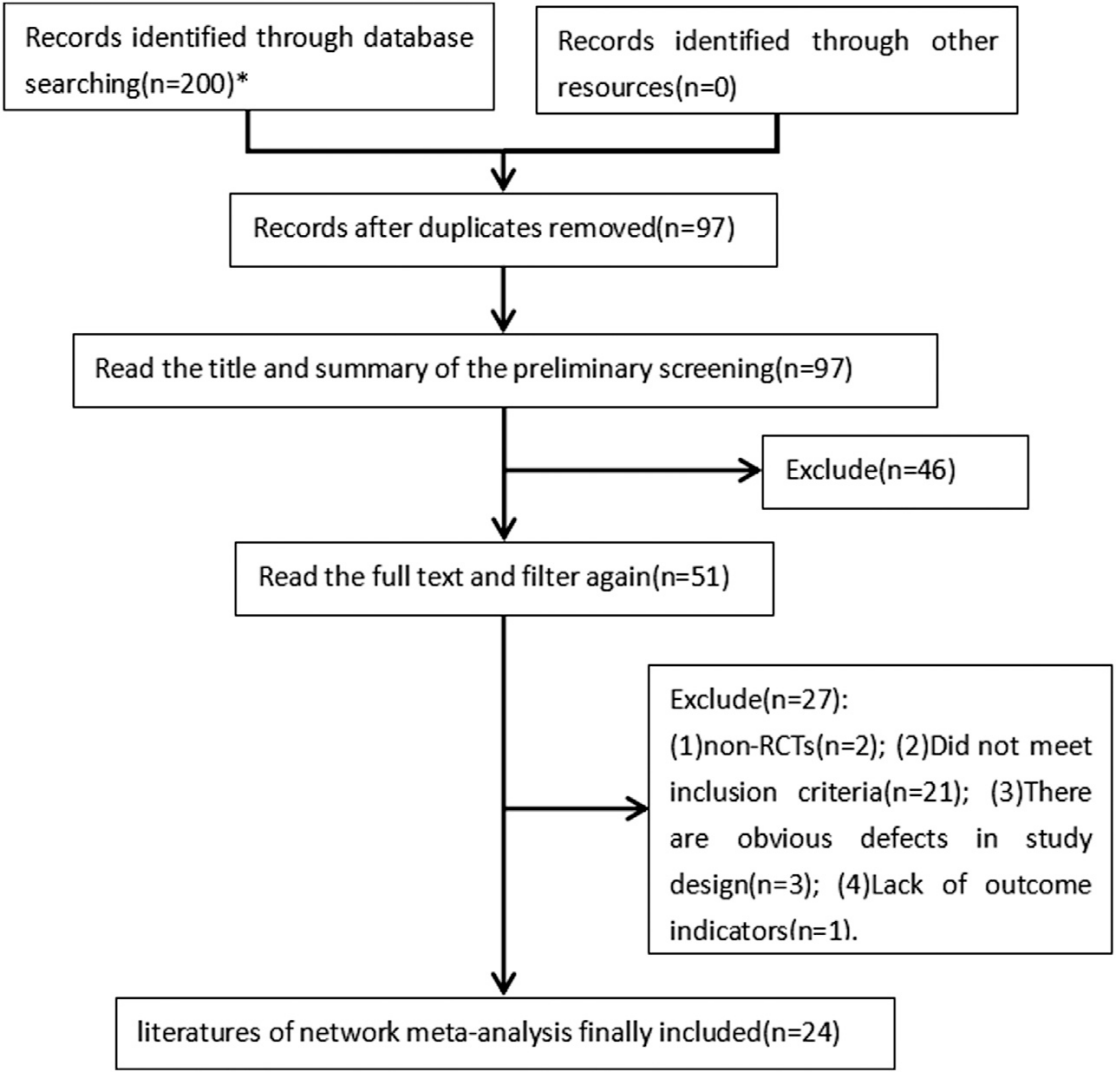
Literature screening process and result diagram. * The databases searched and the number of articles retrieved are as follows: China National Knowledge Infrastructure (*n* = 42), Viper (n = 27), Wanfang (*n* = 41), Chinese Biomedical Literature (*n* = 46), PubMed (*n* = 17), Embase (*n* = 10), and The Cochrane Central Register of Controlled Trials (*n* = 17).

A total of 200 related articles were obtained through the initial examination. After screening layer by layer, 24 RCTs (Ji et al., 2015; Jian and Bai, 2016; Yang and Huang, 2016; Yang and Zhou, 2016; Chen, 2017; Liu, 2017; Hao and Xia, 2018; Ma, 2018; Wang et al., 2018; Yu et al., 2018; Zhang, 2018; Zhang et al., 2018; Zhong et al., 2018; Chen, 2019; Liu, 2019; Wang, 2019; Yang et al., 2019; Zhong et al., 2019; Chu et al., 2020; Fan and Xue, 2020; Li, 2020; Liang et al., 2020; Liu, 2020; Wei et al., 2020) were finally included, all of which were Chinese literature and involved a total of 2,318 cases. The literature screening process and results are shown in Figure 1.

### Basic Features of the Included Studies

**FIGURE 2:**
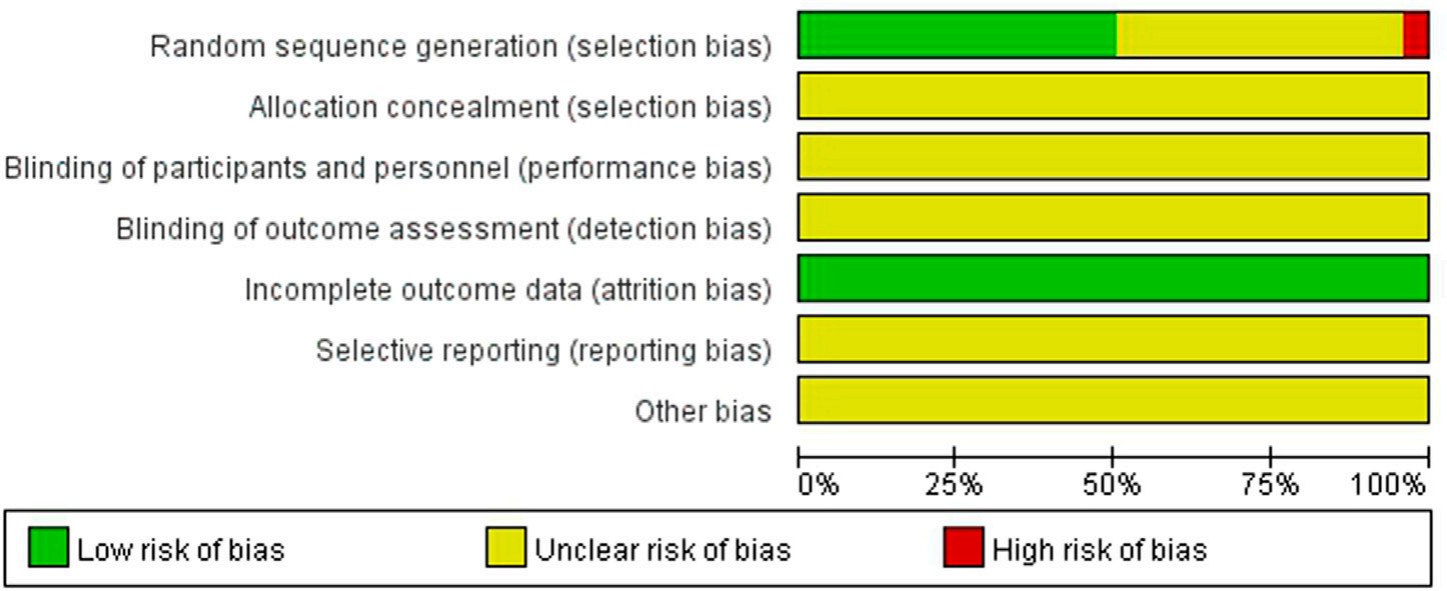
Results of the bias risk assessment of the included studies.

The subjects of this study were patients with ovulation disorders, all of whom were from studies that involved double-arm clinical trials, and a total of six kinds of Chinese patent medicine preparations were finally included from 15 studies of Kuntai capsules, two studies of Fuke Zaizao capsules, two studies of Fufang Xuanju capsules, 1 study of Fuke Yangying capsules, 1 study of Bailing capsules, and three studies of Dingkun Dan. Of these studies, 12 reported the ovulation rate (1,045 cases), 15 reported the pregnancy rate (1,442 cases), and 21 reported the endometrial thickness on the day of follicular maturity (2,070 cases) after treatment. The basic features of the included studies are shown in Table 1, and the assessment results of the risk of bias tests are shown in Figure 2.

**TABLE 1:** Basic features of the included studies.

No.	Liter-ature code	Number of included cases		Age(year, $\bar{\text{x}}\pm \text{s}$ )		Course of disease (months, $\bar{\text{x}}\pm \text{s}$ )		Base-line comparability	Treatment measures and dosage	Control measures and doses	Course	Out-come
		Trial group	Control group	Trial group	Control group	Trial group	Control group					
1	Yang and Huang (2016)	35	35	36.4 ± 2.3	36.2 ± 2.1	25 ± 1.1	24 ± 1.1	comparable	KT 2 g tid po + LE	LE 2.5 mg qd po, ×7D, Menstruation D5 begins	50 days	③
2	Yang and Zhou (2016)	82	80	26.44 ± 3.67	26.53 ± 3.77	not mentioned	not mentioned	comparable	KT 2 g tid po + LE	LE 2.5 mg qd po, ×5D, Menstruation D3 begins	4 weeks	②③
3	Chen (2017)	40	40	not mentioned	not mentioned	not mentioned	not mentioned	comparable	KT 2 g tid po + LE	LE 2.5 mg qd po, ×7D, Menstruation D5 begins	50 days	③
4	Wang et al. (2018)	55	50	not mentioned	not mentioned	not mentioned	not mentioned	comparable	KT 2 g tid po + LE	LE 2.5 mg qd po,×5D, Menstruation D5 begins	Until preg-nancy or mens-trua-tion	②③
5	Yu et al. (2018)	42	42	29.42 ± 2.34	29.11 ± 2.28	24.45 ± 1.67	24.32 ± 1.41	comparable	KT 2 g tid po + LE	LE 2.5 mg qd po, ×5D, Menstruation D5 begins	30 days	①②③
6	Zhang (2018)	52	52	28.35 ± 3.12	28.18 ± 3.88	not mentioned	not mentioned	comparable	KT 2 g tid po + LE	LE 2.5 mg qd po,×5D, Menstruation D5 begins	4 weeks	②③
7	Zhong et al. (2018)	60	60	not mentioned	not mentioned	not mentioned	not mentioned	comparable	KT 2 g tid po + LE	LE 2.5 mg qd po,×7D, Menstruation D5 begins	50 days	③
8	Chen (2019)	40	40	34.5 ± 3.2	32.5 ± 3.6	27.6 ± 6	26.4 ± 9.6	comparable	KT 2 g tid po + LE	LE 2.5 mg qd po,×5D, Menstruation D5 begins	50 days	③
9	Liu (2019)	46	46	28.75 ± 2.59	29.17 ± 2.66	25.68 ± 15	26.88 ± 16.56	comparable	KT 2 g tid po + LE	LE 2.5 mg qd po, ×5D, Menstruation D5 begins	30 days	①②
10	Wang (2019)	48	48	31.23 ± 3.09	31.04 ± 3.21	23.04 ± 6.36	22.44 ± 5.76	comparable	KT 2 g tid po + LE	LE 2.5 mg qd po, ×5D, Menstruation D5 begins	3 months	③
11	Yang et al. (2019)	55	55	29.59 ± 8.12	29.74 ± 7.19	38.76‬±10.32	38.16 ± 9	comparable	KT 2 g tid po + LE	LE 2.5 mg qd po, ×5D, Menstruation D5 begins	3 months	①②③
12	Zhong et al. (2019)	45	45	28.61 ± 3.11	28.56 ± 3.14	13.11‬±1.61	13.09 ± 1.56	comparable	KT 2 g tid po + LE	LE 2.5 mg qd po, ×5D, Menstruation D5 begins	6 months	①②③
13	Fan and Xue (2020)	46	46	33.96 ± 8.12	33.21 ± 7.38	27‬±8.28	26.28 ± 9.36	comparable	KT 2 g tid po + LE	LE 2.5 mg qd po, ×5D, Menstruation D5 begins	3 months	③
14	Li (202)	50	50	34.56 ± 4.12	34.75 ± 3.51	not mentioned	not mentioned	comparable	KT 2 g tid po + LE	LE 2.5 mg qd po, ×5D, Menstruation D5 begins	3 months	①②③
15	Liu (2020)	60	60	29.68 ± 2.77	30.01 ± 2.21	not mentioned	not mentioned	comparable	KT 2 g tid po + LE	LE 2.5 mg qd po, ×5D, Menstruation D5 begins	3 months	②③
16	Jian and Bai (2016)	45	44	29.05 ± 5.36	29.11 ± 5.01	72.6‬±12.24	74.16 ± 13.8	comparable	ZZ 6 pills bid po + LE	LE 2.5 mg qd po, ×5D, Menstruation D5 begins	2 months	①②③
17	Liu (2017)	48	48	31.7 ± 3.3	31.8 ± 3.5	not mentioned	not mentioned	comparable	ZZ 6 pills bid po + LE	LE 2.5 mg qd po, ×5D, Menstruation D5 begins	2 months	①②
18	Hao and Xia (2018)	75	75	28.97 ± 4	29.02 ± 4.01	not mentioned	not mentioned	comparable	XJ 3 pills tid po + LE	LE 2.5 mg qd po, ×5D, Menstruation D3 begins	Until preg-nancy or mens-trua-tion	③
19	Liang et al. (2020)	44	50	29.25 ± 5.28	30.22 ± 6.43	not mentioned	not mentioned	comparable	XJ 3 pills tid po + LE	LE 2.5 mg qd po, ×5D, Menstruation D3 begins	3 months	①③
20	Ji et al. (2015)	30	30	25.4 ± 3.78	23.7 ± 4.34	not mentioned	not mentioned	comparable	YY 4 pills tid po + LE	LE 2.5 mg qd po, ×5D, Menstruation D5 begins	4 months	①②
21	Zhang et al. (2018)	47	47	35.61 ± 6.03	34.85 ± 6.12	22.44‬±11.4	22.08 ± 1.32	comparable	BL 1 pills tid po + LE	LE 2.5 mg qd po, ×5D, Menstruation D5 begins	3 months	③
22	Ma (2018)	40	40	26.2 ± 4	25.4 ± 4.2	42‬±13.2	44.4 ± 14.4	comparable	DK 0.5–1 pill bid po + LE	LE 2.5 mg qd po, ×5D, Menstruation D3-5 begins	3 months	①②③
23	Chu et al. (2020)	30	30	29.27 ± 3.59	29.17 ± 3.51	not mentioned	not mentioned	comparable	DK 1 pill bid po + LE	LE 2.5 mg qd po, ×5D, Menstruation D3-5 begins	1 month	①②③
24	Wei et al. (2020)	45	45	26.12 ± 3.54	27.35 ± 3.29	not mentioned	not mentioned	comparable	DK 1 pill bid po + LE	LE 2.5–5 mg qd po, ×5D, Menstruation D3-5 begins	3 months	①②③

P.S.: Outcome indicators:①Ovulation rate; ②Pregnancy rate; ③Endometrial thickness on the follicular maturity day.

KT, Kuntai capsule; LE, letrozole; ZZ, Fuke Zaizao capsule; XJ, Fufang Xuanju Capsule; YY, Fuke Yangying capsule; BL,Bailing capsule; DK, Dingkun Dan.

### Pairwise Meta-Analysis

The results of the pairwise meta-analysis showed that when compared with patients using only letrozole, the ovulation rate was higher in patients using letrozole combined with Kuntai capsules, Fuke Zaizao capsules, Fufang Xuanju capsules, or Dingkun Dan (*α* < 0.05), but there was no significant difference in patients using Fuke Yangying capsules combined with letrozole (*α* > 0.05); the pregnancy rate was higher in patients using letrozole combined with Kuntai capsules, Fuke Zaizao capsules, or Dingkun Dan (*α* < 0.05), but there was no significant difference in patients using Fuke Yangying capsules combined with letrozole (*α* > 0.05); and the endometrial thickness on the day of follicular maturity was greater in patients using letrozole combined with Kuntai capsules, Fuke Zaizao capsules, Fufang Xuanju capsules, Bailing capsules, or Dingkun Dan (*α* < 0.05). The results of the pairwise meta-analysis are shown in Table 2.

**TABLE 2:** Results of pairwise meta-analysis.

Interven-tions	Included studies	OR/MD(95%CI)	P	Z	P	I^2^ (%)	Tau^2^
Ovulation rate							
KT + LE vs LE	5	3.52(2.06, 6.04)	0.0000	4.59	0.94	0	
ZZ + LE vs LE	2	8.30(2.76, 25.00)	0.0002	3.76	0.96	0	
XJ + LE vs LE	1	20.24(2.55, 160.32)	0.004	2.85			
YY + LE vs LE	1	1.80(0.39, 8.32)	0.45	0.75			
DK + LE vs LE	3	4.06(2.09, 7.91)	0.0000	4.12	0.79	0	
Pregnancy rate							
KT + LE vs LE	9	2.41(1.80, 3.22)	0.0000	5.94	0.81	0	
ZZ + LE vs LE	2	1.98(1.06, 3.72)	0.03	2.13	0.98	0	
YY + LE vs LE	1	1.41(0.45, 4.45)	0.56	0.58			
DK + LE vs LE	3	3.76(2.15, 6.60)	0.0000	4.62	0.99	0	
Endometrial thickness on the follicular maturity day							
KT + LE vs LE	14	2.27(1.70, 2.84)	0.0000	7.84	0.0000	94	0.97
ZZ + LE vs LE	1	2.23(1.56, 2.90)	0.0000	6.49			
XJ + LE vs LE	2	3.35(3.08, 3.62)	0.0000	24.14	0.60	0	0.00
BL + LE vs LE	1	1.16(0.38, 1.94)	0.004	2.91			
DK + LE vs LE	3	1.63(0.58, 2.68)	0.002	3.05	0.0000	91	0.77

### Network Meta-Analysis

#### Evidence Network

**FIGURE 3:**
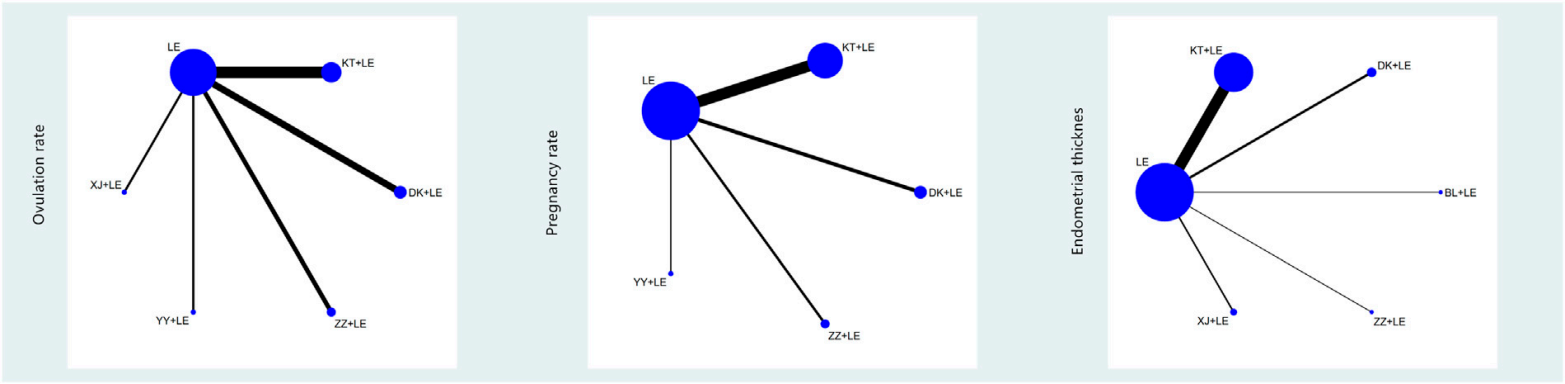
Evidence chart of the ovulation rate/pregnancy rate/endometrial thickness in the network meta-analysis of the different Chinese patent medicines combined with letrozole in the treatment of ovulation disorders.

Three outcome indicators were considered, i.e., ovulation rate, pregnancy rate, and endometrial thickness on the day of follicular maturity. Star structure charts were created with letrozole as the center of the star structure. There were six intervention nodes in the ovulation rate star structure chart, five in the pregnancy rate star structure chart, and six in the endometrial thickness on the day of follicular maturity star structure chart. No closed loop was formed in any of the above evidence networks (see Figure 3).

### Model Selection

When the results of the random-effect model are similar to those of the fixed-effect model, the data can be considered to be stable. The DICs of the ovulation rate and pregnancy rate were smaller in the fixed-effect model, and the DIC for endometrial thickness on the day of follicular maturity was smaller in the random-effect model (see Table 3). Therefore, the fixed-effect model of the dichotomous variables (ovulation and pregnancy rates) and the random-effect model of the continuous variable (endometrial thickness on the day of follicular maturity) were used for data analysis in this study.

**TABLE 3:** DIC values of fixed effect model and random effect model.

Interventions	Model	Dbar	Dhat	PD	DIC
Ovulation rate	Fixed effect model	102.841	85.965	16.876	119.717
	Random effect model	103.551	85.334	18.217	121.768
Pregnancy rate	Fixed effect model	145.785	126.701	19.084	164.868
	Random effect model	146.389	125.675	20.714	167.103
Endometrial thickness on the follicular maturity day	Fixed effect model	227.026	201.020	26.006	253.033
	Random effect model	−7.126	−46.902	39.777	32.651

### Heterogeneity Test

None of the three outcome indicators in this study exhibited a closed loop, so a consistency test was not necessary.

#### Evaluation of the Small Sample Effect

**FIGURE 4:**
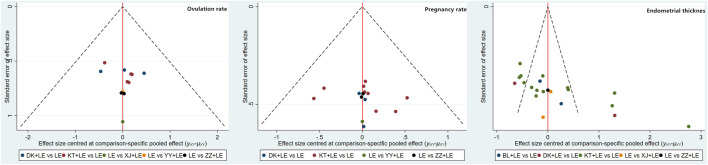
Funnel plot of the comparison correction for ovulation rate/pregnancy rate/endometrial thickness.

The comparison-correction funnel plot of the ovulation and pregnancy rates showed that all the studies were distributed symmetrically around the X = 0 line, and none of the studies fell outside of the funnel plot, indicating that there was no evidence of a small sample effect in the research network. However, in the comparison-correction funnel plot of the endometrial thickness on the day of follicular maturity, not all studies were symmetrically distributed around the X = 0 line, and nine studies were located outside the funnel plot, which provides evidence for the small sample effect in the research network (see Figure 4).

#### Results of the Network Meta-Analysis of the Three Outcome Indicators

##### Ovulation Rate

A total of 12 studies (Ji et al., 2015; Jian and Bai, 2016; Liu, 2017; Ma, 2018; Yu et al., 2018; Liu, 2019; Yang et al., 2019; Zhong et al., 2019; Chu et al., 2020; Li, 2020; Liang et al., 2020; Wei et al., 2020) reported the ovulation rate after treatment, which was higher in patients using letrozole combined with Kuntai capsules, Fuke Zaizao capsules, Fufang Xuanju capsules, or Dingkun Dan than in those using only letrozole and was significantly higher in patients using Fufang Xuanju capsules combined with letrozole than in patients using the other combinations, but there was no statistically significant difference between the other treatment results (see Table 4).

**TABLE 4:** Results of network meta-analysis of different Chinese patent medicines combined with letrozole in the treatment of ovulation disorders.

Ovulation rate									
KT + LE									
3.185 (0.7724, 9.7730)	ZZ + LE								
43.050 (1.1800, 212.10)	19.3000 (0.3382, 93.360)		XJ + LE						
0.8054 (0.0990, 3.1900)	0.3585 (0.0281, 1.5770)		0.1520 (0.0019, 0.8321)		YY + LE				
1.1670 (0.4391, 2.5550)	0.5175 (0.1018, 1.4970)		0.2197 (0.0049, 0.9483)		2.9500 (0.3124, 11.190)		DK + LE		
0.2858 (0.1580, 0.4700)	0.1267 (0.0312, 0.3111)		0.0540 (0.0014, 0.2164)		0.7232 (0.0928, 2.5160)		0.2772 (0.1289,0.5121)		LE
**Pregnancy Rate**									
KT + LE									
0.8738 (0.4091, 1.659)		ZZ + LE							
0.7115 (0.1755, 2.0040)		0.9039 (0.1886, 2.7570)		YY + LE					
1.6670 (0.8427, 3.0010)		2.1190 (0.8246, 4.5030)		3.3800 (0.7190, 9.961)		DK + LE			
0.4131 (0.3048, 0.5471)		0.5252 (0.2623, 0.9342)		0.8370 (0.2108, 2.2490)		0.2693 (0.1454, 0.4514)		LE	
**Endometrial Thickness on the Follicular Maturity Day**									
KT + LE									
0.0385 (−2.2780, 2.391)	ZZ + LE								
−1.0940 (−2.747, 0.5871)	−1.1330 (−3.8560, 1.5800)		XJ + LE						
1.1100 (−1.2340, 3.4920)	1.0720 (−2.1140, 4.2830)		2.2040 (−0.5356, 4.9510)		BL + LE				
0.6452 (−0.7600, 2.0890)	0.6067 (−1.9830, 3.2090)		1.7390 (−0.2594, 3.7510)		−0.4649 (−3.073, 2.151)		DK + LE		
2.2720 (1.661, 2.9160)	2.2340 (−0.0146, 4.4850)		3.3660 (1.8270, 4.9150)		1.1620 (−1.1200,3.437)		1.6270 (0.3441, 2.9080)		LE

##### Pregnancy Rate

A total of 15 studies (Ji et al., 2015; Jian and Bai, 2016; Yang and Zhou, 2016; Liu, 2017; Ma, 2018; Wang et al., 2018; Yu et al., 2018; Zhang, 2018; Liu, 2019; Yang et al., 2019; Zhong et al., 2019; Chu et al., 2020; Li, 2020; Liu, 2020; Wei et al., 2020) reported the pregnancy rate after treatment, which was higher in patients using letrozole combined with Kuntai capsules, Fuke Zaizao capsules, or Dingkun Dan that in those using only letrozole,but there was no statistically significant difference between the other treatment protocols (see Table 4).

##### Endometrial Thickness on the Day of Follicular Maturity

A total of 21 studies (Jian and Bai, 2016; Yang and Huang, 2016; Yang and Zhou, 2016; Chen, 2017; Hao and Xia, 2018; Ma, 2018; Wang et al., 2018; Yu et al., 2018; Zhang, 2018; Zhang et al., 2018; Zhong et al., 2018; Chen, 2019; Wang, 2019; Yang et al., 2019; Zhong et al., 2019; Chu et al., 2020; Fan and Xue, 2020; Li, 2020; Liang et al., 2020; Liu, 2020; Wei et al., 2020) reported the endometrial thickness on the day of follicular maturity after treatment, which was higher in patients using letrozole combined with Kuntai capsules, Fufang Xuanju capsules, or Dingkun Dan than in those using only letrozole. There was no statistically significant difference between the other treatment protocols (see Table 4).

#### Sequencing of Network Meta-Analysis Results

The relative ranking results of the three outcome indicators were different (a lower average ranking is better). Based on the three outcome indicators, the intervention effect of the Fufang Xuanju capsules combined with letrozole was the greatest in terms of increasing the ovulation rate and the endometrial thickness on the day of follicular maturity. In terms of increasing the pregnancy rate, the intervention effect of Dingkun Dan combined with letrozole was the greatest (see Table 5).

**TABLE 5:** Sequencing results of network meta-analysis of different Chinese patent medicines combined with letrozole in the treatment of ovulation disorders.

Interventions	Ovulation rate		Pregnancy rate		Endometrial thickness on the follicular maturity day	
	WinBugs results	Rank	WinBugs results	Rank	WinBugs results	Rank
KT + LE	0.0015	5	0.0560	3	0.0420	3
ZZ + LE	0.1596	2	0.0493	4	0.1726	2
XJ + LE	0.8261	1	-	-	0.7344	1
YY + LE	0.0089	3	0.0622	2	-	-
BL + LE	-	-	-	-	0.0343	4
DK + LE	0.0040	4	0.8325	1	0.0167	5
LE	0.0000	6	0.0000	5	0.0000	6

## Discussion

### Discussion on the Components and Pharmacological Effects of Chinese Patent Medicines Involved in This Study

In this study, except for the Bailing capsule, which is a single plant extract, the other five Chinese patent medicines are all compound preparations. These five types of Chinese patent medicines mainly contain the following traditional Chinese medicines:rehmanniae radix praeparata (shu di) , paeoniae radix alba (bai shao), asini corii colla (e jiao), poria (fu ling), angelicae sinensis radix (dang gui), cyperi rhizoma (xiang fu), atractylodis macrocephalae rhizoma (bai shu), eucommiae cortex (du zhong), chuanxiong rhizoma (chuan xiong), leonuri herba (yi mu cao), and scutellariae radix (huang qin). See Supplementary Table S1 for detailed composition information.

Modern pharmacological studies have found that rehmanniae radix praeparata, paeoniae radix alba, and scutellariae radix have anti-inflammatory, antioxidant, and immune-regulating effects. Poria and atractylodis macrocephalae rhizoma can regulate blood glucose and lipid metabolism. Paeoniae radix alba, asini corii colla, cyperi rhizoma, and leonuri herba have estrogen-like effects, which can improve ovarian function and regulate the contraction activity of the uterine smooth muscle. Angelicae sinensis radix has an anticoagulant and antithrombotic function and can improve the microcirculation of the reproductive system. Polyrhachis vicina roger is the main component of the Fufang Xuanju capsule, and studies have found that it can coordinate the endocrine system in the body, regulate the synthesis of hormones, and boost immunity. It can also effectively regulate blood sugar and blood lipid levels in the body. The Bailing capsule contains fermented cordyceps powder, which has the effect of regulating immunity and the glucose metabolism of the body.

The composition of Chinese patent medicine is complex, with many drug targets, and the mechanism of the pharmacological action is not completely clear. At present, the main results of the pharmacological research on the six Chinese patent medicines in the treatment of ovulation disorders are as follows:

Bailing capsules can effectively improve glucose metabolism and reduce the degree of insulin resistance in patients, which are important mechanisms in improving ovulation disorders. (Liu et al., 2018)

Fufang Xuanju capsules can stimulate the production of endometrial blood vessels by regulating angiogenic factors, which improves uterine blood circulation, thereby improving the nutritional status of the endometrium. By regulating the nitric oxide levels, it can relieve vascular smooth muscle contractions and improve endometrial blood flow to promote the implantation of the embryo. In addition, the compound Xuanju capsule contains selenium, zinc, and other trace elements, which can effectively regulate the hypothalamic–pituitary–ovarian–uterus axis. (Huang et al., 2016; Zhang and Yu, 2017)

The Kuntai capsule can improve ovarian function by upregulating the level of superoxide dismutase and regulating the expression of the B-cell lymphoma 2 (Bcl-2) and Bcl-2-associated X proteins through the oxidative stress pathway (Geng and Tan, 2017; Zhang et al., 2017) and can also regulate the level of norepinephrine and 5-hydroxytryptamine, which improves ovarian function by regulating the hypothalamic–pituitary–ovarian–uterus axis. (Duan et al., 2014; Zhang et al., 2016)

The Fuke Zaizao capsule can regulate the level of androgens in patients with polycystic ovary syndrome and inhibit the expression of microRNA (miRNA)-383 and miRNA-320 in follicular granulosa cells, which regulates the expression of downstream target genes and hormone synthesis and produces the corresponding biological effect. (Han, 2017)

The Fuke Yangying capsule can effectively improve ovarian blood circulation, promote the development and maturation of dominant follicles, promote ovulation, improve luteal function, and improve endometrial receptivity to increase pregnancy and embryo survival rates. (Xie et al., 2012)

Dingkun Dan has an estrogen-like effect, (Li et al., 2016) can dilute cervical mucus during the period surrounding ovulation, is conducive to sperm penetration, can improve ovarian function, can promote follicle development, and can increase the ovulation rate. (Zhai, 2019) In addition, it can promote endometrial growth, improve endometrial receptivity, and increase the pregnancy rate in patients struggling with infertility. (Huang, 2018)

### Mechanism of Ovulation Induction by Letrozole

Ovulation disorders are the main cause of infertility or difficulty achieving pregnancy in women of childbearing age who require fertility treatment, which seriously affects women’s physical and mental health and family harmony. Ovulation induction is the main treatment for ovulation disorders, and letrozole, a third-generation aromatase inhibitor, is currently commonly used to promote ovulation. When compared with clomiphene, a first-line ovulatory drug, letrozole has been shown to reduce the risk of ovarian hyperstimulation syndrome, increase the endometrial thickness, and improve the cervical mucus score. (Yu Q. et al., 2019; Behnoud et al., 2019)

### The Possible Pharmacological Effects of Chinese Patent Medicine and Letrozole on Ovulation Induction

Although letrozole has many advantages over clomiphene for ovulation induction, numerous clinical studies have found that due to the short half-life of the drug, the effect of letrozole decreases continuously as estrogen levels drop in the later stage of follicular development, resulting in the possibility that the dominant follicles may not be released. Various studies have shown that the success rate of letrozole for ovulation induction is about 70–84%, but the pregnancy rate is only 20–27%. (Yu Z. Z. et al., 2019)

According to the pharmacological effects of the abovementioned Chinese patent medicines, the author believes that Chinese patent medicine can achieve complementary effects with letrozole, such as regulating glucose metabolism, alleviating insulin resistance, improving endometrial blood circulation, promoting endometrial growth, improving endometrial receptivity, regulating hormone levels and the reproductive axis, and improving the conception rate, thus improving the efficacy of letrozole in treating ovulation disorders.

### The Feasibility of Using Chinese Patent Medicine in Combination With Letrozole in the Treatment of Ovulation Disorders

In traditional Chinese medicine theory, the kidney dominates reproduction: “… kidney dominates Chong and Ren meridian, Chong meridian is the repository of blood, Ren meridian connects with the uterus and pregnancy.” Therefore, there is a close relationship between fertility and both kidney deficiency and the disharmony of the qi and blood of zang-fu. Tonifying the kidney and filling essence and harmonizing the qi and blood are the main methods to treat infertility. Doctors in Western hospitals and general hospitals in China are more inclined to use Chinese patent medicine to treat ovulation disorders when they are combined with ovulation promoting medicines. The Chinese patent medicines in this study, which tonify the kidney and harmonize the qi and blood, are recognized by the majority of front-line doctors in clinical practice.

This study evaluated the efficacy of six Chinese patent medicines combined with letrozole in the treatment of ovulation disorders. The results showed that the combination of Fufang Xuanju capsules and letrozole was more effective than the other intervention measures in improving the ovulation rate and endometrial thickness on the day of follicular maturity, and the combination of Dingkun Dan and letrozole was better than other the intervention measures in improving the pregnancy rate.

### The Innovations and Limitations of This Study

Chinese patent medicines are widely used on the clinical front line, especially by gynecologists and reproductive doctors in general hospitals. In this study, the maximum number of citations for a single Chinese patent medicine pharmacological study was 115 times, and the maximum number of citations for a single article in the literatures included in the NMA was 38 times (See Supplementary Table S2 for specific citation times). It can be seen that Chinese patent medicine has always been a hot topic in Medical research in China. However, in the past, there has been little comparison of efficacy and professional guidelines for various Chinese patent medicines.

This study is part of the project of Guidelines on clinical application of Chinese patent medicines Standardized Program. This project was set up by the State Administration of Traditional Chinese Medicine, and dozens of general hospitals in first-tier cities across the country participated in it. By looking for evidence-based medicine evidence and combining expert clinical drug use experience, expert consensus was finally reached, and guidelines were formed and issued to guide clinical front-line doctors to use Chinese patent medicines. This is of great significance for reducing clinical risk and improving clinical efficacy.

In previous studies, there was a lack of comparison of efficacy of many proprietary Chinese medicines. In this paper, the advantages and disadvantages of six proprietary Chinese medicines in the treatment of ovulation disorders were analyzed, providing a reference for clinical front-line doctors, and laying a foundation for the development of relevant guidelines in the next step.

Of course, there are still limitations: 1) The quality of the included research is low. Some of the literature did not clarify the random method, and all of the literature lacked an appropriate blinding method, so the results of this NMA are likely to be biased to some extent. The potential risks associated with this bias may affect the authenticity and reliability of the results and lead to reduced test performance. 2) The sample size of the included literature is small, and the statistical efficacy may be insufficient. 3) The quality of results from the NMA was not graded in this study. Based on the defects of the existing research, the decision makers should consider the influence of the above factors and consider carefully when applying the research conclusions.

Therefore, there is a need for relevant studies in the future to further optimize the design scheme of clinical trials. It is expected that future studies can further guide the rational use of drugs in the clinical setting.

## Conclusion

Through network meta-analysis, we demonstrated that Letrozole combined with Chinese patent medicine was more effective than letrozole alone in the treatment of ovulation disorders. Fufang Xuanju capsules was good at improving the ovulation rate and increasing the endometrial thickness. Dingkun Dan was good at improving the pregnancy rate. These founding can provide an important reference for clinicians in specific medication.

## Data Availability

The raw data supporting the conclusions of this article will be made available by the authors, without undue reservation.
