# Supplementary material for: An Efficacy and Feasibility Analysis of Chinese Patent Medicine Combined With Letrozole in the Treatment of Women With Ovulation Disorders: A Network Meta-Analysis
Source: Front Pharmacol. 2021 Oct 5;12:722122. doi: 10.3389/fphar.2021.722122 (PMC8524095; doi:10.3389/fphar.2021.722122)
Supplement: Supplementary file 2 [file Table3.doc]

Supplement table: Pubmed is retrieved.

| Search number | Query | Search Details | Results |
| --- | --- | --- | --- |
| 1 | chinese medicine | ("chin med"[Journal] OR ("chinese"[All Fields] AND "medicine"[All Fields])) OR "chinese medicine"[All Fields] | 209,529 |
| 2 | herbal medicine | ("herbal medicine"[MeSH Terms] OR ("herbal"[All Fields] AND "medicine"[All Fields])) OR "herbal medicine"[All Fields] | 46,527 |
| 3 | patent medicine | ((("nonprescription drugs"[MeSH Terms] OR ("nonprescription"[All Fields] AND "drugs"[All Fields])) OR "nonprescription drugs"[All Fields]) OR ("patent"[All Fields] AND "medicine"[All Fields])) OR "patent medicine"[All Fields] | 26,982 |
| 4 | #1 OR #2 OR #3 | ((("chin med"[Journal] OR ("chinese"[All Fields] AND "medicine"[All Fields])) OR "chinese medicine"[All Fields]) OR (("herbal medicine"[MeSH Terms] OR ("herbal"[All Fields] AND "medicine"[All Fields])) OR "herbal medicine"[All Fields])) OR (((("nonprescription drugs"[MeSH Terms] OR ("nonprescription"[All Fields] AND "drugs"[All Fields])) OR "nonprescription drugs"[All Fields]) OR ("patent"[All Fields] AND "medicine"[All Fields])) OR "patent medicine"[All Fields]) | 249,808 |
| 5 | letrozole | "letrozole"[MeSH Terms] OR "letrozole"[All Fields] OR "letrozol"[All Fields] | 3,292 |
| 6 | #4 AND #5 | (((("chin med"[Journal] OR ("chinese"[All Fields] AND "medicine"[All Fields])) OR "chinese medicine"[All Fields]) OR (("herbal medicine"[MeSH Terms] OR ("herbal"[All Fields] AND "medicine"[All Fields])) OR "herbal medicine"[All Fields])) OR (((("nonprescription drugs"[MeSH Terms] OR ("nonprescription"[All Fields] AND "drugs"[All Fields])) OR "nonprescription drugs"[All Fields]) OR ("patent"[All Fields] AND "medicine"[All Fields])) OR "patent medicine"[All Fields])) AND (("letrozole"[MeSH Terms] OR "letrozole"[All Fields]) OR "letrozol"[All Fields]) | 68 |
| 7 | Kuntai | "Kuntai"[All Fields] | 57 |
| 8 | bailing | "bailing"[All Fields] | 67 |
| 9 | xuanju | "xuanju"[All Fields] | 18 |
| 10 | dingkun | "dingkun"[All Fields] | 12 |
| 11 | yangrong | "yangrong"[All Fields] | 13 |
| 12 | fuke zaizao | "fuke"[All Fields] AND "zaizao"[All Fields] | 1 |
| 13 | #7 OR #8 OR #9 OR #10 OR #11 OR #12 | (((("Kuntai"[All Fields] OR "bailing"[All Fields]) OR "xuanju"[All Fields]) OR "dingkun"[All Fields]) OR "yangrong"[All Fields]) OR ("fuke"[All Fields] AND "zaizao"[All Fields]) | 167 |
| 14 | #13 OR #4 | ((((("Kuntai"[All Fields] OR "bailing"[All Fields]) OR "xuanju"[All Fields]) OR "dingkun"[All Fields]) OR "yangrong"[All Fields]) OR ("fuke"[All Fields] AND "zaizao"[All Fields])) OR (((("chin med"[Journal] OR ("chinese"[All Fields] AND "medicine"[All Fields])) OR "chinese medicine"[All Fields]) OR (("herbal medicine"[MeSH Terms] OR ("herbal"[All Fields] AND "medicine"[All Fields])) OR "herbal medicine"[All Fields])) OR (((("nonprescription drugs"[MeSH Terms] OR ("nonprescription"[All Fields] AND "drugs"[All Fields])) OR "nonprescription drugs"[All Fields]) OR ("patent"[All Fields] AND "medicine"[All Fields])) OR "patent medicine"[All Fields])) | 249,917 |
| 15 | #14 AND #5 | (((((("Kuntai"[All Fields] OR "bailing"[All Fields]) OR "xuanju"[All Fields]) OR "dingkun"[All Fields]) OR "yangrong"[All Fields]) OR ("fuke"[All Fields] AND "zaizao"[All Fields])) OR (((("chin med"[Journal] OR ("chinese"[All Fields] AND "medicine"[All Fields])) OR "chinese medicine"[All Fields]) OR (("herbal medicine"[MeSH Terms] OR ("herbal"[All Fields] AND "medicine"[All Fields])) OR "herbal medicine"[All Fields])) OR (((("nonprescription drugs"[MeSH Terms] OR ("nonprescription"[All Fields] AND "drugs"[All Fields])) OR "nonprescription drugs"[All Fields]) OR ("patent"[All Fields] AND "medicine"[All Fields])) OR "patent medicine"[All Fields]))) AND (("letrozole"[MeSH Terms] OR "letrozole"[All Fields]) OR "letrozol"[All Fields]) | 68 |
| 16 | #14 AND #5 | (((((("Kuntai"[All Fields] OR "bailing"[All Fields]) OR "xuanju"[All Fields]) OR "dingkun"[All Fields]) OR "yangrong"[All Fields]) OR ("fuke"[All Fields] AND "zaizao"[All Fields])) OR (((("chin med"[Journal] OR ("chinese"[All Fields] AND "medicine"[All Fields])) OR "chinese medicine"[All Fields]) OR (("herbal medicine"[MeSH Terms] OR ("herbal"[All Fields] AND "medicine"[All Fields])) OR "herbal medicine"[All Fields])) OR (((("nonprescription drugs"[MeSH Terms] OR ("nonprescription"[All Fields] AND "drugs"[All Fields])) OR "nonprescription drugs"[All Fields]) OR ("patent"[All Fields] AND "medicine"[All Fields])) OR "patent medicine"[All Fields]))) AND (("letrozole"[MeSH Terms] OR "letrozole"[All Fields]) OR "letrozol"[All Fields]) Filters: Clinical Trial, Meta-Analysis, Randomized Controlled Trial, Systematic Review | 17 |
